# Rule-Based Non-Intrusive Load Monitoring Using Steady-State Current Waveform Features

**DOI:** 10.3390/s23156926

**Published:** 2023-08-03

**Authors:** Hussain Shareef, Madathodika Asna, Rachid Errouissi, Achikkulath Prasanthi

**Affiliations:** Electrical and Communication Engineering Department, United Arab Emirates University, Al Ain 15551, United Arab Emirates; shareef@uaeu.ac.ae (H.S.); asna.m@uaeu.ac.ae (M.A.); prasanthi@uaeu.ac.ae (A.P.)

**Keywords:** current waveform, event detection, feature extraction, load identification, non-intrusive load monitoring, set theory

## Abstract

Monitoring electricity energy usage can help to reduce power consumption considerably. Among load monitoring techniques, non-intrusive load monitoring (NILM) provides a cost-efficient solution to identify individual load consumption details from the aggregate voltage and current measurements. Existing load monitoring techniques often require large datasets or use complex algorithms to obtain acceptable performance. In this paper, a NILM technique using six non-redundant current waveform features with rule-based set theory (CRuST) is proposed. The architecture consists of an event detection stage followed by preprocessing and framing of the current signal, feature extraction, and finally, the load identification stage. During the event detection stage, a change in connected loads is ascertained using current waveform features. Once an event is detected, the aggregate current is processed and framed to obtain the event-causing load current. From the obtained load current, the six features are extracted. Furthermore, the load identification stage determines the event-causing load, utilizing the features extracted and the appliance model. The results of the CRuST NILM are evaluated using performance metrics for different scenarios, and it is observed to provide more than 96% accuracy for all test cases. The CRuST NILM is also observed to have superior performance compared to the feed-forward back-propagation network model and a few other existing NILM techniques.

## 1. Introduction

Based on the modern day loads used by the general public, electrical energy is expected to eventually replace all other forms of end-use energy [1]. Therefore, understanding the specifics of electrical energy usage is essential for increasing energy efficiency, conserving energy, and reducing emissions [2]. The electricity consumption details basically comprise information on each electrical appliance’s consumption, including operating state, power demand, total electricity energy, and fault diagnosis. This information can help in increasing energy efficiency and power grid asset management [3,4]. From the end user’s perspective, the information on electricity consumption can encourage them to become energy efficient and thereby result in potential energy savings [5,6]. Hence, an energy efficiency solution built on load monitoring techniques can help reduce electricity consumption considerably [7]. The methods and techniques for monitoring electric energy usage and providing appropriate feedback on usage patterns to users can be briefly classified as intrusive and non-intrusive. Despite the accuracy and dependability of the data acquired, the intrusive method has difficulty in terms of its early implementation, need for expensive hardware, and low user approval [8]. On the other hand, the non-intrusive load monitoring (NILM) method, uses less equipment because there are fewer components to install, making it a convenient and affordable method of load monitoring [9,10].

The existing NILM approaches can be briefly classified from the problem perspective as optimization-based problems, pattern recognition problems and rule-based classification problems. The optimization algorithm finds the best combination of state of the appliances through different randomization strategies and iterative processes. NILM was first developed as a combinatorial optimization problem [11], but this approach has a major shortcoming when multiple loads of similar power ratings are required to be identified. To tackle this issue, many improved optimization techniques have been developed. In Ref. [12], the NILM algorithm was formulated as a least square error minimization problem to identify the active appliances according to the observed power profile. Similar to this, Ref. [13] developed a NILM system based on particle swarm optimization (PSO) and integer programming (IP), by extracting the features of the power signal. However, the exponential complexity of these algorithms limits their use in real-time cases.

On the other hand, the NILM approach as a pattern recognition problem predominantly uses clustering and classification algorithms such as an artificial neural network [14,15], a convolutional neural network [16], and diffusion models [17]. The above-mentioned machine-learning-based methods can be used to overcome the difficulty of distinguishing between similar rated loads by utilizing the features from the training data [18]. However, when machine learning is employed, large sets of data are required to develop an accurate appliance model. Further re-training would be required when the load set changes, and therefore, the method is not easily transferable. Even though unsupervised learning methods have been developed to enable the transferability of models [19], the requirement of large datasets still remain a limitation. In addition, machine-learning-based algorithms predict the state of each appliance during every sampling period, which increases the computational cost as the number of appliances to be predicted increases. In addition, these algorithms are generally applied to disaggregate high-power appliances (e.g., the air conditioning), whereas the error rapidly increases for low-power appliances.

In this regard, using event-based NILM algorithms where events are detected and classified using certain rules provides a finer granularity at lower computational cost than eventless machine-learning-based NILM algorithms [20,21]. In addition, event-based techniques require fewer datasets to develop the appliance models and also the noise and measurement errors have a reduced effect on their performance. The predominant shortcomings of existing rule-based NILM techniques are the dependency on a single signature and the effect of noise and measurement errors on it. When a single load signature is used for event detection and load identification, NILM may result in the non-identification or false identification of loads, specifically when the signature has a low signal-to-noise ratio. To reduce the effect of noise and errors, many NILM methods specify a threshold to avoid false event detection and identification of loads [22]. However, the value of this threshold is a trade-off between the smallest change in the considered load signature and the maximum possible measurement error. This is particularly difficult when the load set consists of loads with a wide range of power ratings. The measurement error can also lead to non-detection of a load change, which is referred to as an event. Additionally, several existing rule-based NILM algorithms employ deep learning techniques [23,24] that highly increase the computational requirements, and optimizing these algorithms can be challenging. The effect of noise due to single load signatures can be overcome by using a rule-based intelligent identification method to distinguish loads with the help of multiple features [25]. The selection of these features requires specific attention to achieve adequate performance. Among the available parameters, the current waveforms are known to be load-specific [26]. Hence, the features of load current waveforms can clearly represent the individual loads, and therefore, their features can help identify connected loads. Furthermore, since an instantaneous load current is periodic in nature, the non-periodic noise and measurement errors can be eliminated by using appropriate filtering techniques.

Considering the above discussion, a NILM technique utilizing current waveform features and rule-based set theory approach (CRuST) is proposed in this work. The CRuST NILM architecture consists of an event detection stage followed by preprocessing and framing of the current signal, the extraction of features, and finally, the load identification stage. In the event detection stage, the change in the event variable, which is the function of root-mean-square (RMS) and total harmonic distortion (THD), is used to identify the occurrence of a change in the connected loads. This reduces the rate of false positive and false negative detections as the event variable value due to noise and the smallest load change is increased. When an event is detected, the aggregate current waveform is processed and framed for a predefined time duration to obtain the current waveform of the load that caused the event. From the preprocessed and framed load current waveform, their features, namely crest factor, form factor, variance w.r.t a sinusoidal reference, RMS, power factor, and THD, are extracted. These features signify and distinguish the waveshapes as well as the power rating of different loads. The proposed NILM architecture also includes the appliance models within which the load sets are classified into subsets for each feature based on the rules of limits defined in their corresponding feature array. These same rules are used in the identification stage to classify and determine the load that caused an event by using a set intersection method. The following assumptions are made:

- Only type 1 loads with ON and OFF states are considered. However, the algorithm shall be further developed to detect type 2 and type 3 loads in future research.
- At any time instant, only one appliance changes its state such that an event is associated with a single appliance.
- The minimum time between two load changes is assumed to be at least one second, so that the load current can be suitably framed for feature extraction.
- The aggregate current at the smart meter is sampled at a frequency large enough to enable accurate feature extraction from the instantaneous aggregate current waveforms.

The submetered time-series current waveform of eight type 1 loads, collected from laboratory experiments, are used as the dataset in this work. The proposed NILM method is tested for multiple scenarios and compared to the actual state of the devices using a confusion matrix. The performance evaluation metrics such as precision, recall, accuracy, individual, macro-averaged, and weighted averaged F-measure, and area under the curve of the receiver operating characteristics are used to evaluate and demonstrate the superior performance of CRuST NILM. The principal contributions of this paper are listed as follows.

1. The requirement of large dataset is eliminated by using a criteria-based approach to classify load-specific current waveform features.
2. A unique appliance model is presented that classifies loads into sets based on feature values in criteria arrays.
3. An improved event detection algorithm, that utilizes two features, each signifying change in current and change in waveshape, is proposed.
4. A novel load identification method using the concept of rule-based set theory is proposed to achieve improved performance.

## 2. Architecture of Proposed CRuST NILM

**Figure 1:**
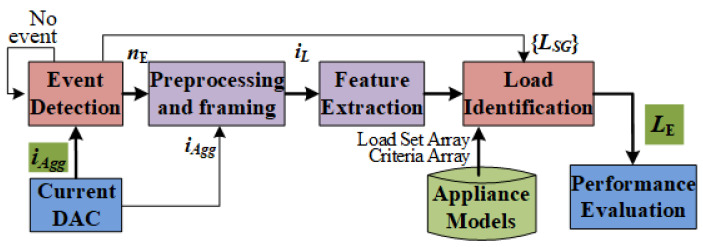
Architecture of proposed CRuST NILM.

The architecture of the CRuST NILM method is presented in Figure 1. The aggregated current waveform $i_{\mathrm{Agg}}$ at real time is obtained from a data acquisition system. The event detection algorithm checks for any changes to the current waveform $i_{\mathrm{Agg}}$. The detection of an event signifies a change in the connected loads. When an event is detected at say instant $n_{E}$, the algorithm waits for a few fundamental cycles of the current waveform to allow its switching transients to settle. The aggregated current $i_{\mathrm{Agg}}$ is then preprocessed and framed. The preprocessing eliminates the noise and measurement errors, while framing separates the current of the load that caused the event from the aggregate load current for the defined frame and window length. The load current waveform $i_{L}$ is then read as a time-series data array from which the current waveform features are extracted, which is further used for load identification. The load identification phase consists of indexing and identifying loads from the search group set $\left\{L_{\mathrm{SG}}\right\}$ with the help of the appliance model.

**Figure 2:**
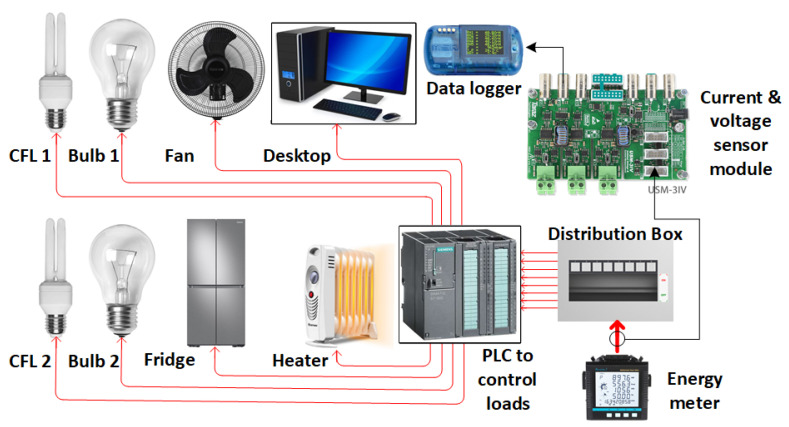
Experimental setup for CRuST NILM.

In the proposed CRuST NILM, the dataset used is a private dataset generated by the research team at the United Arab Emirates University laboratory. The experimental setup used to generate the dataset is shown in Figure 2, which includes six different type −1 appliances, namely two compact fluorescent lamps (CFL), two incandescent light bulbs, a table fan, a desktop computer, a fridge, and a room heater.

**Figure 3:**
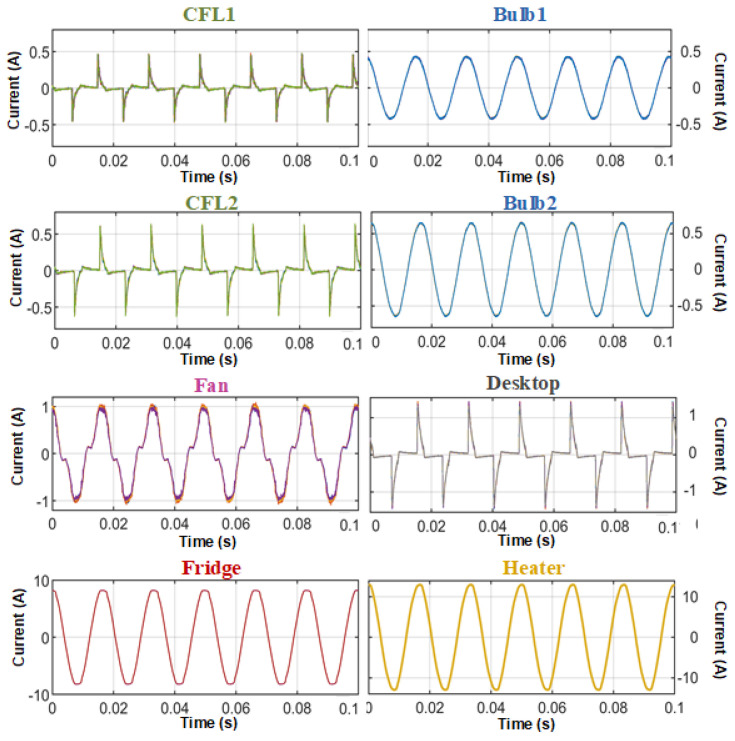
Load current waveforms of selected loads.

The current signals for each load are then measured and the processed submetered data for these loads are presented in Figure 3. In order to identically apply feature extraction methods to create an appliance model, the time-series data samples of these loads are preprocessed by phase shifting and time clipping appropriately. Here, six features pertaining to the load current waveforms are considered for load identification.

For effective load identification, the choice of features of each of the loads should be unique and non-redundant. Hence, in this paper, the crest factor, form factor, and variance of the normalized harmonic components of the load current w.r.t. normalized sinusoidal waveform are used in addition to the root-mean-square (RMS) of the fundamental component, power factor, and THD of the load current. Among these, the RMS and power factor values of the current signify the power consumption of the loads, the variance and THD represent a certain aspect of the waveform shape, while the crest factor and form factor includes both the current magnitude as well as its waveshape information. These six features are obtained for two random datasets of each of the eight loads as listed in Table 1 and are used to develop the appliance model for a load feature library that envelops several of the important features that signify and distinguish the current waveforms of different waveforms.

### 2.1. Appliance Models

**Figure 4:**
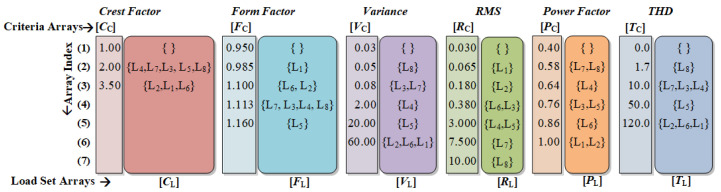
Classification of load set in load library based on criteria array.

The appliance model of the proposed CRuST NILM consists of six criteria arrays and load set arrays constructed based on the six considered features as shown in Figure 4. For instance, for the feature *C*, i.e., crest factor, the appliance model includes the criteria array and load set array [$C_{C}$] and [$C_{L}$], while for *F*, i.e., form factor, the model incorporates [$F_{C}$] and [$F_{L}$], and so on. The criteria arrays hold the upper limits for the features based on which the loads are grouped into sets in the corresponding load set array. The selection of the values for the criterion array and the grouping in the load set arrays for the loads of Table 1 are further detailed as follows.

#### 2.1.1. Criteria Array

From Table 1, it can be observed that each load has a unique combination of values for the features. Thus, a set of rules called separation criteria is developed based on the range of values of loads’ features. Six criteria array, namely $\left[C_{C}\right]$, $\left[F_{C}\right]$, $\left[V_{C}\right]$, $\left[R_{C}\right]$, $\left[P_{C}\right]$, and $\left[T_{C}\right]$ are defined. The upper separation limits for each feature are listed in its corresponding criteria array. For example, the crest factor of the current waveform of $L_{7}$ (Fridge) is 0.964, while that of $L_{8}$ (Heater) is 0.993. To classify $L_{7}$ and $L_{8}$ under different sets in the load set array [$C_{L}$], the criteria array [$C_{C}$] should specify the separation criteria 0.97 and 1.00. Similar separation criteria are followed for the rest of the loads to form the criteria array for each of the features. When the values of a feature for two or more loads are comparable, a common upper criteria is chosen.

#### 2.1.2. Load Set Array

Consider the load set *L* that includes all the eight loads labeled $L_{1}$ to $L_{8}$, i.e., $L=\{L_{1},L_{2},L_{3},L_{4},L_{5},L_{6},L_{7},L_{8}\}$. To relate each load to the separation criterion, the load set *L* is grouped into several subsets based on the values of corresponding features. The classification of these subsets is performed based on the separation criteria specified in the criteria array. Each of the six features relate to one load set array, and are termed $\left[C_{L}\right]$, $\left[F_{L}\right]$, $\left[V_{L}\right]$, $\left[R_{L}\right]$, $\left[P_{L}\right]$, and $\left[T_{L}\right]$. Under each load set array, the loads are grouped based on the separation criteria specified in the corresponding index of the criteria array. Consider the feature crest factor, C, for the loads $L_{1}$ to $L_{8}$. If the crest factor of the load current of $L_{i}$ is less than $C_{C}\left(x_{r}-1\right)$ but greater than $C_{C}\left(x_{r}\right)$, then $L_{i}$ will be listed in the ${\left(x_{r}-1\right)}^{}$th row of the load set array [$C_{L}$], i.e., in set $C_{L}\left(x_{r}-1\right)$. Similarly, each of the loads are categorized and listed based on the values in the criteria array. Since only type 1 loads are considered in this paper, each load is listed only once in a load set array. A null set within a load set array signifies there are no existing loads that fall under the corresponding separation criterion.

The process of the classification of loads in the load set array can be further detailed using the following case. An if−else algorithm to classify these loads based on the crest factor values *C*($L_{i}$) is presented in Algorithm 1. Here, $C_{L}\left(j\right)=C_{L}\left(j\right)\cup$$L_{i}$ is used to include the load $L_{i}$ to the load set in the $j^{}$th index of the load set array $C_{L}$.
**Algorithm 1** Defining load set array1:**for** i←1 to 8 **do**2:   **for** j←1 to length($C_{C}$) **do**3:     **if** $C_{C}\left(j-1\right)<C\left(L_{i}\right)<C_{C}\left(j\right)$ **then**4:        $C_{L}\left(j\right)=C_{L}\left(j\right)\cup L_{i}$5:     **end if**6:   **end for**7:**end for**

The load set arrays are developed for the rest of the features in a similar manner. This forms the appliance model, which is further used for load identification.

### 2.2. Event Detection

**Figure 5:**
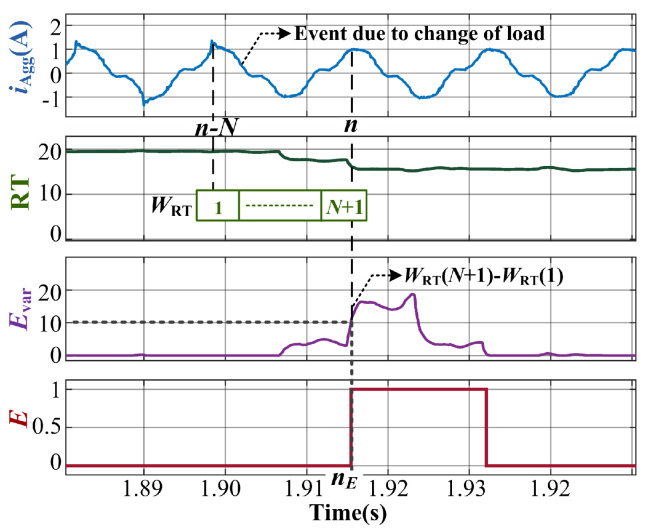
Waveforms corresponding to event detection using RMS and THD values of the aggregate load current $i_{\mathrm{Agg}}$.

An event is termed as the occurrence of a change in the connected loads signified by the change in power or current. Thus, it can be identified by the change in the aggregate current $i_{\mathrm{Agg}}$ as shown in Figure 5. The effect of noise and measurement error make it difficult to identify an event because many of the low power loads such as CFL, bulb, and desktop computer cause very low change in the power measurement in comparison with those such as fridge and heater. Furthermore, the errors and noises in the measurements for the high-power devices and the power or current values of the low-power devices are of comparable magnitudes. In this case, a certain NILM algorithm utilizes transient features to determine an event. However, these may fail when the change in loads causes considerably low transient effects, such as that shown in Figure 5. These can lead to false detection when events are detected only by using power measurements. On the other hand, using a combination of non-redundant features can aid in a more accurate event detection.

In the proposed CRuST NILM method, event detection is performed by observing the RMS and THD of the aggregate current waveform $i_{\mathrm{Agg}}$. The RMS value indicates change in power level, while THD incorporates the harmonics and indicates the change in the waveform shape. To quantify the change in these variables, their difference w.r.t. $N_{c}$ fundamental cycle is considered. For a current waveform $i_{\mathrm{Agg}}$ of fundamental frequency $f_{g}$ and sampled at $f_{s}$, the number of samples in one fundamental cycle is $N=\frac{f_{s}}{f_{g}}$. Thus the delay length $N_{d}$ corresponding to $N_{c}$ fundamental cycles is determined by (1).

(1) $$N_{d}=N_{c}N$$

Considering one fundamental cycle, i.e., $N_{c}=1$, the event detection variable $E_{\mathrm{var}}$ is determined as in (2).

(2) $$\begin{array}{c} E_{\mathrm{var}}=f\left(R,T\right)={\left(RT\left(n\right)-RT\left(n-N\right)\right)}^{2} \end{array}$$

Here, *R* and *T* are the RMS and THD of the current waveform, respectively. RT(n) and RT(n−N) are the products of RMS and THD at present sampling instant *n* and one fundamental cycle prior to instant *n* respectively.

To implement (2), a moving window, $W_{\mathrm{RT}}$, of length N+1, is used. From Figure 5, at any instant *n*, $W_{\mathrm{RT}}\left(1\right)=RT\left(n-N\right)$ and $W_{\mathrm{RT}}\left(N+1\right)=RT\left(n\right)$. The event detection variable can be obtained as $E_{\mathrm{var}}={\left(W_{\mathrm{RT}}\left(N+1\right)-W_{\mathrm{RT}}\left(1\right)\right)}^{2}$. The window moves forward by one sample at a sampling frequency same as the current waveform, thereby enabling the variable computation in real time.

An event *E* is signified by a change in the variable $E_{\mathrm{var}}$ by a magnitude greater than *e* as expressed by (3). Here, *e* accounts for the noise and measurement errors and $n_{E}$ is the sampling instant at which the event is observed to occur.

(3) $$\begin{array}{cccc} E(n) & =1, & n_{E}=n; & \mathrm{if}\;E_{\mathrm{var}}\left(n\right)>e\\ \; & =0; & & otherwise \end{array}$$

Based on the values for $E_{\mathrm{var}}$ observed for various combinations of loads mentioned in Table 1, the margin for error is 3≤e≤14. Hence, *e* can be safely fixed as 10 as shown in Figure 5. The slight delay between the actual occurrence of an event and the detected event *E* is negligible as time between two consecutive events is considerably more (at least few seconds) in comparison to this delay. The above-described event detection method is expected to have superior performance over the conventional method of using power-related variations, especially when the considered loads have a wide range of power ratings. The chances of false positive event detection due to transients is avoided by using a counter to help discard successive events within a predefined duration to allow transients to settle down.

To reduce the search space, when an event is detected, the load search group $L_{\mathrm{SG}}$ is defined based on whether a new load was switched ON or an existing load was turned OFF using the RMS values as presented in Algorithm 2. To avoid the effect of transients, the values of RMS are considered one fundamental cycle before the event and 5 fundamental cycles after. Here, $L_{\mathrm{on}}\left(n_{E}\right)$ and $L_{\mathrm{off}}\left(n_{E}\right)$ denote the set of loads that were identified to be ON and OFF, respectively, prior to the event occurrence at the sampling instant $n_{E}$. Note that since $L_{\mathrm{on}}$ and $L_{\mathrm{off}}$ are non-intersecting subsets of the load set *L*, at any instant *n*, $L=L_{\mathrm{on}}\left(n\right)\cup L_{\mathrm{off}}\left(n\right)$.
**Algorithm 2** Defining load search group1:**if** $R(n_{E}+5N)$ > $R(n_{E}-N)$ **then**2:   $L_{\mathrm{SG}}=L_{\mathrm{off}}\left(n_{E}\right)$3:**else**4:   $L_{\mathrm{SG}}=L_{\mathrm{on}}\left(n_{E}\right)$5:**end if**

**Figure 6:**
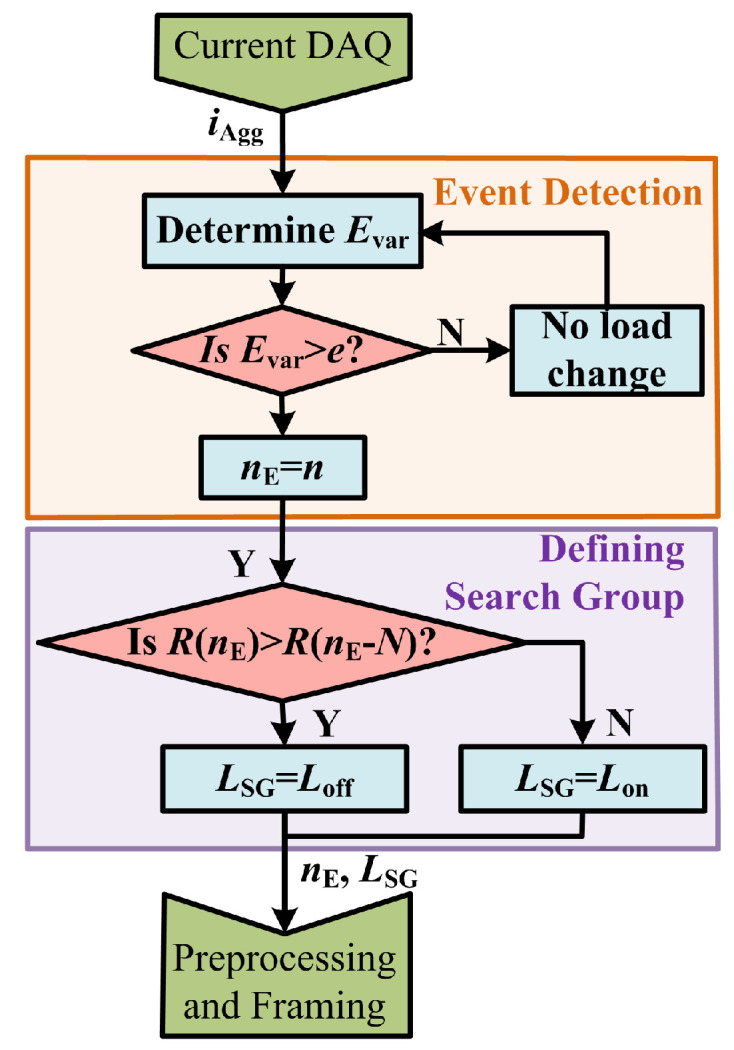
Flowchart of event detection and search group definition.

The flowchart representing the event detection and defining the search group is presented in Figure 6. The instant of event occurrence, $n_{E}$, is passed on to preprocessing and framing stage where the current waveform is used for feature extraction and the load search group $L_{\mathrm{SG}}$ is used for the load identification to reduce the search size.

### 2.3. Preprocessing and Framing

When an event is detected, the load current waveform features are expected to be extracted and further used for the identification of the load that caused the event. Since the features are required to be extracted from individual load currents, the load current that caused the event, $i_{L_{E}}$, should be separated from the aggregate load current $i_{\mathrm{Agg}}$ while avoiding the effects of transients due to the load change. In the loads listed in Table 1, all the current waveforms are observed to attain steady-state within $\Delta t_{\mathrm{tr}}=$ 0.3 s. Furthermore, for the purpose of feature extraction, the load current waveform is framed for $\Delta t_{f}=$ 0.5 s, and an additional delay time of $\Delta t_{\mathrm{ad}}=$ 0.05 s is considered to avoid overlapping of transients. Thus, the event-causing load current $i_{L_{E}}$ can be characterized as in (4), where the total time difference is $\Delta t_{d}=\Delta t_{\mathrm{tr}}+\Delta t_{f}+\Delta t_{\mathrm{ad}}=0.85$.

(4) $$i_{L_{E}}\left(t\right)=i_{\mathrm{Agg}}\left(t\right)-i_{\mathrm{Agg}}\left(t-\Delta t_{d}\right)$$

In discrete-time domain, the number of samples $N_{d}$ corresponding to the delay $\Delta t_{d}$ can be determined as in (5).

(5) $$N_{d}=\Delta t_{d}f_{s}$$

**Figure 7:**
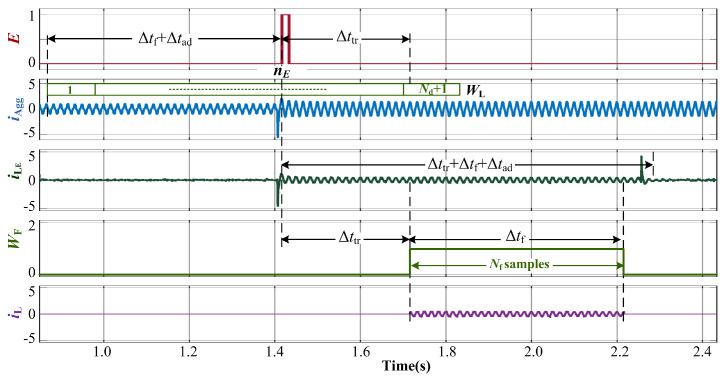
Waveforms corresponding to preprocessing and framing stage to obtain load current $i_{L}$.

To implement (4), and thereby obtain $i_{L_{E}}\left(n\right)$, a moving window, $W_{E_{L}}$, of length $N_{d}+1$, is used. The window moves forward by one sample at a frequency equivalent to the sampling frequency $f_{s}$ of the current waveform. Therefore, the load current at any sampling instant *n* is $i_{L_{E}}\left(n\right)=W_{L}\left(N_{d}+1\right)-W_{L}\left(1\right)$ as shown in Figure 7.

The number of samples, $N_{f}$, corresponding to framing duration, $\Delta t_{f}$, is obtained by $N_{f}=\Delta t_{f}\times f_{s}=$ 15,000 samples in the discrete-time domain. A rectangular window $W_{F}$ of unit magnitude and length $N_{f}$ positioned $\Delta t_{\mathrm{tr}}=$ 0.3 s after E→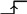, is used for framing $i_{L_{E}}$ as shown in Figure 7. Thus, the framed current $i_{L}$ is obtained using (6).

(6) $$i_{L}=W_{F}i_{L_{E}}$$

The framed current waveform $i_{L}$ corresponds to the load that caused the event at $n_{E}$. This load current waveform is used as time-series data in the feature extraction stage.

### 2.4. Feature Extraction

**Figure 8:**
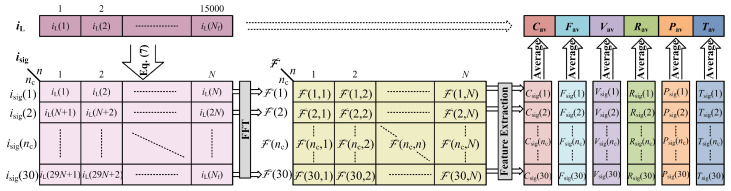
Separation of $i_{L}$ into fundamental cycles as $i_{\mathrm{sig}}$ for feature extraction.

From the preprocessed and framed current signal, the six features mentioned in Table 1 are extracted. The framed signal, $i_{L}$, is a time-series data array with sample length of $N_{f}$ samples. Hence, there are $N_{c}=\frac{N_{f}}{N}=\frac{15,000}{500}=$ 30 fundamental cycles. For a more accurate feature extraction, the features are computed for each fundamental cycle and the values are averaged for 30 cycles. Each fundamental cycle in the time-series array $i_{L}$ is separated in $i_{\mathrm{sig}}$ as shown in Figure 8 using (7), where $1\leq n_{c}\leq N_{c}$. The resulting matrix $i_{\mathrm{sig}}$ will have a size of $N_{c}$-by-N = 30-by-500.

(7) $$i_{\mathrm{sig}}\left(n_{c},n\right)=i_{L}\left(n+(n_{c}-1)N\right)$$

The features crest factor, form factor, variance, RMS, power factor, and THD values are denoted as $C_{\mathrm{sig}}$, $F_{\mathrm{sig}}$, $V_{\mathrm{sig}}$, $R_{\mathrm{sig}}$, $P_{\mathrm{sig}}$, and $T_{\mathrm{sig}}$, respectively. Each feature variable is a column matrix of length *N* as shown in Figure 8 and their corresponding average values are denoted by suffix ’av’. The following processes and equations are employed for the purpose of feature extraction from each row of $i_{\mathrm{sig}}$.

Fast Fourier Transform (FFT) can provide information on the magnitude, frequency, phase, and harmonics of a signal, which are useful for the computation of the aforementioned features. Let the N-point FFT of $n_{c}^{\mathrm{th}}$ row of signal $i_{\mathrm{sig}}$, i.e., $i_{\mathrm{sig}}\left(n_{c}\right)$, be represented by $F(n_{c})$, which is an array of length N=500 as shown in Figure 8. The elements of the array $F(n_{c})$ signify the magnitude and phase of the corresponding harmonic components of the $i_{\mathrm{sig}}\left(n_{c}\right)$. Hence, the magnitude and phase of the fundamental frequency component of $i_{\mathrm{sig}}\left(n_{c}\right)$ can be deduced as (8) and (9), respectively, where $F_{r}\left(n_{\mathrm{ci}},2\right)$ and $F_{i}\left(n_{c},2\right)$ are the real and imaginary parts of $F(n_{c},2)$.

- Magnitude of fundamental frequency component
  (8) $$I_{1}\left(n_{c}\right)=\left|F\left(n_{c}\right)\right|=\sqrt{F_{r}{\left(n_{c},2\right)}^{2}+F_{i}{\left(n_{c},2\right)}^{2}}$$
- Phase of fundamental component
  (9) $$\theta_{1}\left(n_{c}\right)=∠F=\tan^{-1}\left(\frac{F_{i}\left(n_{c},2\right)}{F_{r}\left(n_{c},2\right)}\right)$$

The values of the features can be calculated with the help of (8) and (9) as follows.

#### 2.4.1. Crest Factor $C_{\mathrm{sig}}$

The crest factor is the ratio of the peak to RMS values of the fundamental component of a signal as shown in (10).

(10) $$C_{\mathrm{sig}}\left(n_{c}\right)=\sqrt{2}\frac{max(i_{\mathrm{sig}}\left(n_{c}\right))}{I_{1}\left(n_{c}\right)}$$

#### 2.4.2. Form Factor $F_{\mathrm{sig}}$

The form factor is the ratio of the RMS of fundamental component of a signal to its average value, represented by (11).

(11) $$F_{\mathrm{sig}}\left(n_{c}\right)=\frac{N}{2\sqrt{2}}\frac{I_{1}\left(n_{c}\right)}{\sum_{k=1}^{N/2}i_{\mathrm{sig}}\left(n_{c}\right)}$$

#### 2.4.3. Variance $V_{\mathrm{sig}}$

Variance is generally defined as the squared standard deviation of a signal from its mean value. However, to obtain a measure that can represent the signal waveform shape, the deviation of signal compared to a reference sinusoidal signal with unity amplitude, $i_{\mathrm{ref}}$, is considered here. The signal $i_{\mathrm{sig}}\left(n_{c}\right)$ is also normalized to unity peak as in (12).

(12) $$i_{\mathrm{sig}}^{n}\left(n_{c}\right)=\frac{i_{\mathrm{sig}}\left(n_{c}\right)}{max(i_{\mathrm{sig}}\left(n_{c}\right))}$$

To avoid the effect of phase shift between the reference sinusoidal signal and $i_{\mathrm{sig}}^{n}$, the variance ratio is computed based the absolute values of FFT. Let $F_{\mathrm{ref}}$ and $F^{n}\left(n_{c}\right)$ be the N-point FFTs of $i_{\mathrm{ref}}$ and $i_{\mathrm{sig}}^{n}$, respectively. The variance percentage can be calculated from the FFT arrays using (13).  

(13) $$V_{\mathrm{sig}}\left(n_{c}\right)=\frac{\sum_{k=1}^{N}{\left(|F_{\mathrm{ref}}\left(n_{c},k\right)\left|-\right|F^{n}\left(n_{c},k\right)|\right)}^{2}}{\sum_{k=1}^{N}{\left|F_{\mathrm{ref}}\left(n_{c},k\right)\right|}^{2}}\times 100$$

#### 2.4.4. RMS $R_{\mathrm{sig}}$

The RMS value of signal can be obtained from the magnitude of the fundamental frequency component as

(14) $$R_{\mathrm{sig}}\left(n_{c}\right)=\frac{I_{1}\left(n_{c}\right)}{\sqrt{2}}$$

#### 2.4.5. Power Factor $P_{\mathrm{sig}}$

The power factor is the cosine of the angle of phase shift between the fundamental components of voltage and current.

(15) $$P_{\mathrm{sig}}\left(n_{c}\right)=\cos\left(\theta_{V1}-\theta_{1}\left(n_{c}\right)\right)$$

Here, $\theta_{V}$ is the phase shift of voltage and $\theta_{1}$ is the phase shift of current obtained from (9).

#### 2.4.6. Total Harmonic Distortion $T_{\mathrm{sig}}$

The THD percentage of the current signal is computed with the help of the FFT signal $F(n_{c})$ as in (16).

(16) $$T_{\mathrm{sig}}\left(n_{c}\right)=\frac{\sqrt{\left(\sum_{k=1}^{\frac{N}{2}}{\left|F\left(n_{c},k\right)\right|}^{2}\right)-F{\left(n_{c},2\right)}^{2}}}{F(n_{c},2)}\times 100$$

The values of features array $C_{\mathrm{sig}}$, $F_{\mathrm{sig}}$, $V_{\mathrm{sig}}$, $R_{\mathrm{sig}}$, $P_{\mathrm{sig}}$, and $T_{\mathrm{sig}}$ are averaged to obtain $C_{\mathrm{av}}$, $F_{\mathrm{av}}$, $V_{\mathrm{av}}$, $R_{\mathrm{av}}$, $P_{\mathrm{av}}$, and $T_{\mathrm{av}}$, respectively, as shown in Figure 8. These values are further used alongside the appliance model and load search group to identify the load that caused the event.

### 2.5. Load Identification Technique

**Figure 9:**
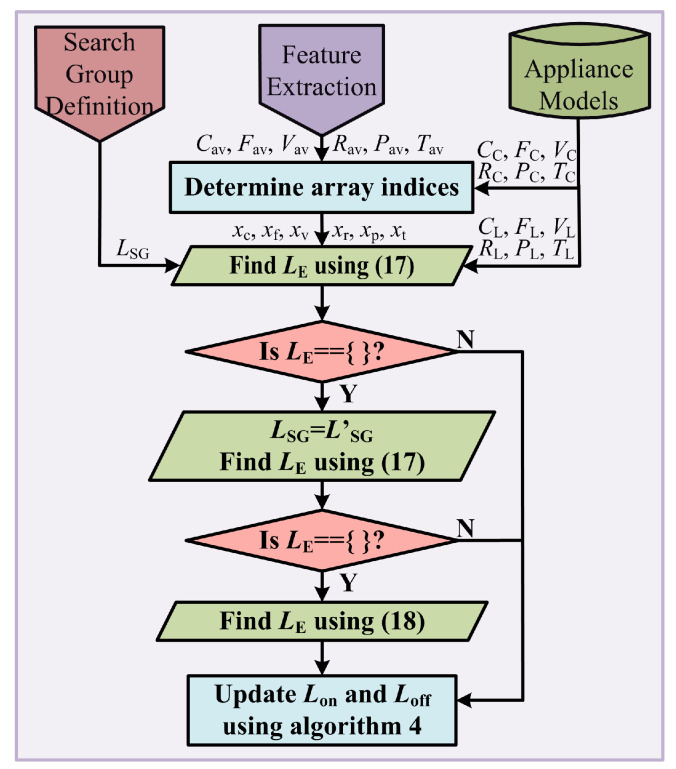
Flowchart of proposed load identification technique.

The proposed load identification technique is demonstrated as a flowchart in Figure 9 and explained in detail as follows.

In the load identification stage, the criteria mentioned in the criteria array of appliance model are used to determine the load that causes the event based on the extracted features. For this purpose, array indices are obtained for which the feature values of the load waveform $i_{L}$ satisfy the criteria in the criteria array. The array indices thus obtained are used to determine the load sets from the load set arrays. The event-causing load is expected within these load sets. For example, say the feature $C_{\mathrm{av}}$ of event-causing load waveform $i_{L}$ satisfies the criteria in $C_{C}\left(x_{c}\right)$, the index $x_{c}$ can be determined by Algorithm 3.
**Algorithm 3** Determine array index1:**for** j←1 to length($C_{C}$) **do**2:   **if** $\frac{C_{C}\left(j\right)}{C_{\mathrm{av}}}\geq 1$ **then**3:     $x_{c}=j$4:     **break**5:   **end if**6:**end for**

The load that caused the event is expected to be within the load set $C_{L}\left(x_{c}\right)$. Similarly, the array indices are determined for the rest of the five features as $x_{f}$, $x_{v}$, $x_{r}$, $x_{p}$, and $x_{t}$. The load corresponding to waveform $i_{L}$ that caused the event can be determined by (17).

(17) $$\begin{array}{c} L_{E}=L_{\mathrm{SG}}\cap C_{L}\left(x_{c}\right)\cap F_{L}\left(x_{f}\right)\cap V_{L}\left(x_{f}\right)\\ \cap R_{L}\left(x_{r}\right)\cap P_{L}\left(x_{p}\right)\cap T_{L}\left(x_{t}\right) \end{array}$$

Since multiple non-redundant features have been used and the search group has been narrowed, (17) yields only one load, which satisfies all the criteria. However, if any of the previous load identifications were incorrect, the load search group would be incorrect. This would result in a null set for $L_{E}$. In such a case, the load search group is modified to $L_{\mathrm{SG}}^{\prime }$, and the event-causing load is recalculated using (17). Furthermore, a null set could still be returned when one or more features are not accurately calculated due to the error in framed load current $i_{L}$. In such a case, the event-causing load is determined as the most repeated load in the combined load set using (18).

(18) $$L_{E}=mode\left(\begin{array}{c} C_{L}\left(x_{c}\right)\cup F_{L}\left(x_{f}\right)\cup V_{L}\left(x_{f}\right)\\ \cup R_{L}\left(x_{r}\right)\cup P_{L}\left(x_{p}\right)\cup T_{L}\left(x_{t}\right) \end{array}\right)$$

Once the event-causing load is determined, the ON and OFF load sets, $L_{\mathrm{on}}$ and $L_{\mathrm{off}}$, are updated based on Algorithm 4. Here, $L_{\mathrm{off}}=L_{\mathrm{off}}\cup L_{E}$ implies the inclusion of load $L_{E}$ to set $L_{\mathrm{off}}$, and $L_{\mathrm{on}}\setminus L_{E}$ implies that $L_{E}$ is removed from the set $L_{\mathrm{on}}$.
**Algorithm 4** Update Load Sets1:**if** $L_{\mathrm{SG}}==L_{\mathrm{off}}$ **then**2:   $L_{\mathrm{off}}=L_{\mathrm{off}}\cup L_{E}$3:   $L_{\mathrm{on}}=L_{\mathrm{on}}\setminus L_{E}$4:**else**5:   $L_{\mathrm{on}}=L_{\mathrm{on}}\cup L_{E}$6:   $L_{\mathrm{off}}=L_{\mathrm{off}}\setminus L_{E}$7:**end if**

## 3. Results and Discussion

The performance of the proposed CRuST NILM is evaluated by comparing the results with the actual conditions for different load switching scenarios using the time-series data obtained from the laboratory. The actual and predicted cases for both event detection as well as load identification are presented as a confusion matrix. As the proposed CRuST NILM depends on the load current, the effect of aggregate current on the event detection and load identification stages is to be evaluated. Therefore, three different scenarios are considered and each of the eight loads are tested for the three scenarios by creating balanced classes.

In the first scenario, a single load is considered to change state at a time, while all others remain in the OFF state. Since there are eight loads, each with an ON and OFF state, there are 16 possible combinations that are considered as an event. On the other hand, for the second scenario, a load change is considered while another load stays ON. Hence, each load has two cases (ON/OFF) with the remaining seven loads. The combination results in 14 cases for one load and therefore, 112 combinations. However, in the test condition, the ON and OFF of individual loads is also considered as an event resulting in an additional 112 combinations. Thus, the total number of combinations for scenario 2 is 224. Similarly, in scenario 3, to test the algorithm at higher aggregate currents, a load change is considered to occur when two loads are in the ON state. Considering all the load changes in such a case, there exists 1008 combinations.

The results are presented as contingency matrices and thereafter used to compute the precision, recall, F-measure, and accuracy for performance evaluation. The precision parameter measures how many of the detected events are actual relevant events, while recall signifies the number of actual relevant events that are detected. The F-measure parameter gives a balance of precision and recall by providing their harmonic mean. Accuracy defines the ratio of the correctly detected events to the total number of events. The results and performance evaluation for the event detection algorithm and load identification technique for the three switching scenarios are discussed further as follows.

### 3.1. Event Detection Algorithm

**Figure 10:**
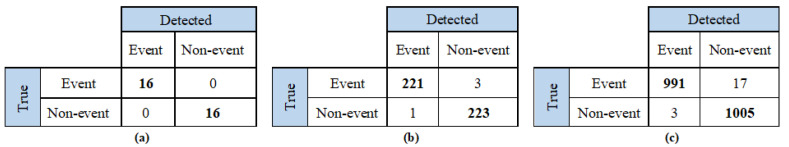
Confusion matrices of event detection algorithm for (**a**) scenario 1 (**b**) scenario 2, and (**c**) scenario 3.

The event detection is evaluated based on the accuracy of detection in the case of an actual event brought about by a change in load using the confusion matrices presented in Figure 10. From the contingency matrices, the performance evaluation metrics for the three test scenarios are computed as shown in Table 2. For the first scenario with a single load operating at a time, the proposed event detection algorithm is capable of detecting all the 16 events accurately. This is as expected, as there is no influence of other loads on the event detection algorithm. Nevertheless, this validates that the effect of noise and measurement noise is minimal in the proposed event detection technique, and hence, all parameter evaluation metrics yield 100% in this scenario.

The results of scenario 2 and 3 can be considered to be more comparable to real-world conditions. Since a balanced class is used and the cost of a false negative is greater than false positive detection, the metrics recall and accuracy are more relevant in comparison. For both these scenarios, the percentage of detected relevant events to the total relevant events, signified by the metric recall, is observed to be greater than 98%. Similarly, the accuracy, i.e., the accurately detected events to the total number of cases, is also computed to be greater than 98% for both the scenarios. Hence, the proposed event detection algorithm can be expected to operate satisfactorily for at least 98% of the cases. This superior accuracy is owing to the use of the event variable that considers change in both RMS and THD of the current waveform.

### 3.2. Load Identification Technique

**Figure 11:**
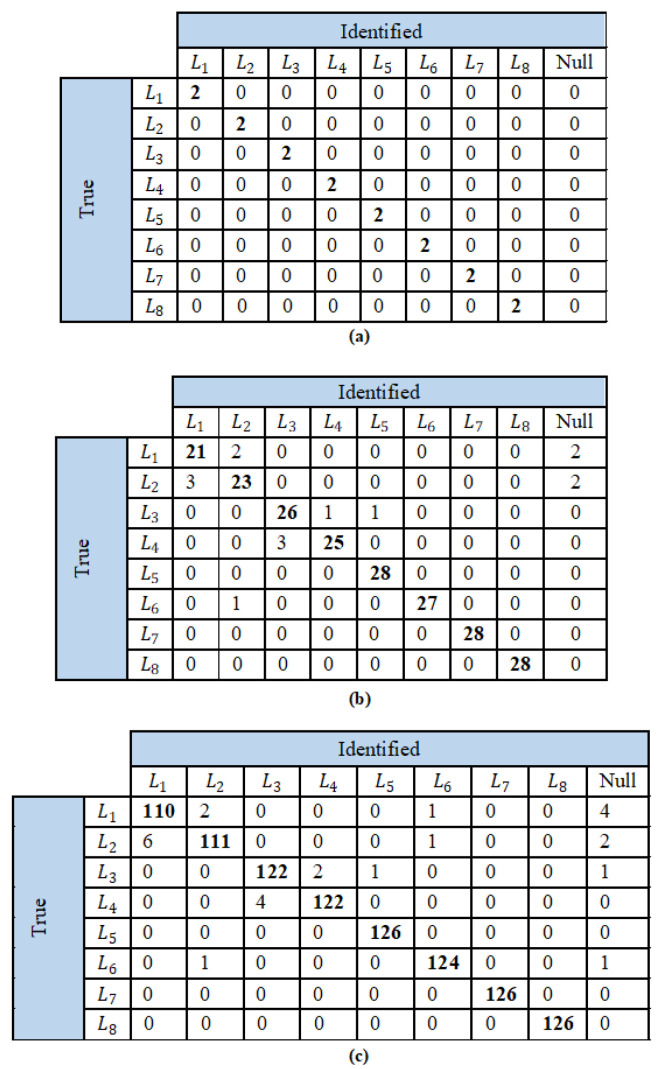
Confusion matrices of load identification technique for (**a**) scenario 1, (**b**) scenario 2, and (**c**) scenario 3.

The proposed load identification technique is assessed using the confusion matrices and the corresponding performance evaluation metrics as presented in Figure 11, and Table 3 and Table 4. Here, only the test cases that returned a true positive in the event detection stage are considered. In the confusion matrix (see Figure 11), the null column indicates that the load identification algorithm failed to relate the extracted features to any of the library load features, and hence returns a null matrix. As with the event detection algorithm, the proposed load identification technique can also accurately classify all the loads in the first scenario. This is because the feature values are correctly computed, while other loads do not influence the framed load current waveform. Therefore, the precision, recall, F-measure, and accuracy calculations yield unity, indicating 100%. However, in the case of scenarios 2 and 3, few loads are not accurately identified. This is because the presence of other loads affects the framed current, and hence its extracted feature values. Nevertheless, the performance evaluation metrics (PEM) are comparable for scenarios 2 and 3. In fact, all the PEMs are observed to improve for the combination of three loads owing to the increased size of the class. The results show accuracy values greater than all the loads, with the least accuracy of identification for $L_{1}$. This is predominantly caused by the narrow difference in features between loads $L_{1}$ and $L_{2}$, both being CFLs, due to which $L_{2}$ is often falsely identified as $L_{1}$ and vice versa. The proposed load identification technique can identify the loads $L_{5}$, $L_{7}$, and $L_{8}$, namely the fan, fridge, and heater, with an accuracy as high as 100%.

### 3.3. Overall Performance Evaluation of CRuST NILM

**Figure 12:**
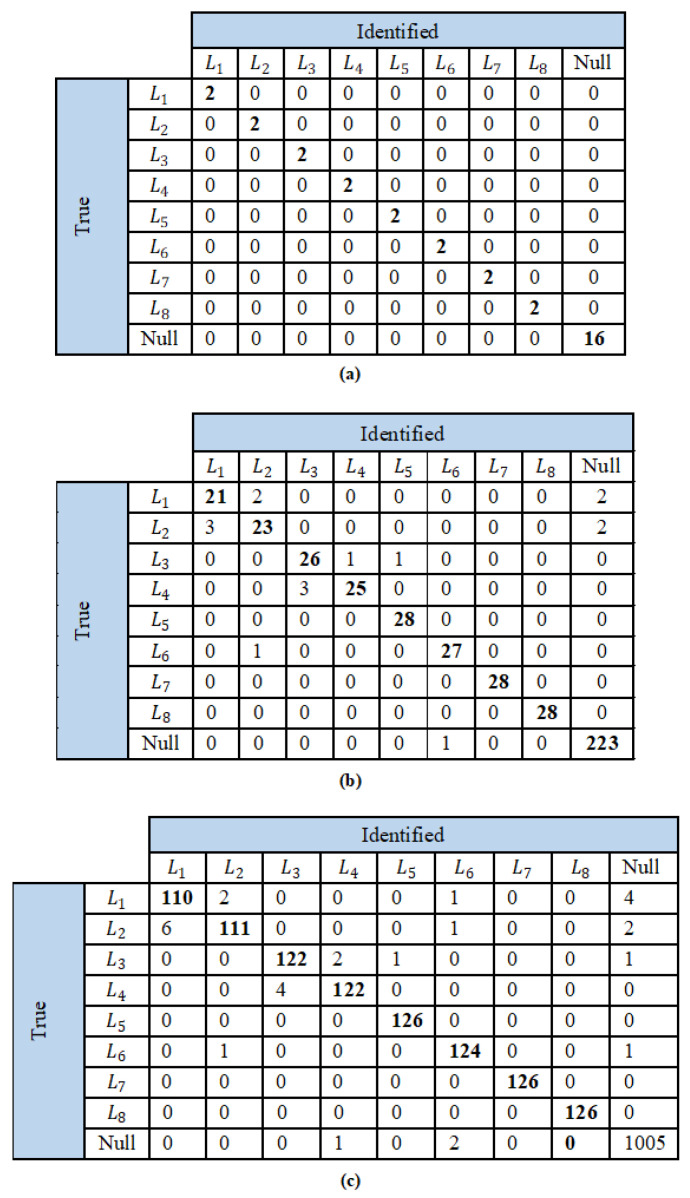
Confusion matrices for the proposed CRuST NILM for (**a**) scenario 1, (**b**) scenario 2, and (**c**) scenario 3.

The overall performance of the proposed CRuST NILM is evaluated considering the same class used in the event detection algorithm. The confusion matrices and the corresponding performance evaluation metrics are presented in Figure 12, and in Table 5 and Table 6. The null row on the ‘true’ side of the confusion matrix given in Figure 12 indicates the loads that were detected and identified while there was no load change; hence, it lists the loads that were identified due to false event detection. Similarly, the null column under the ‘identified’ list indicates the loads identified during false negative event detection as well as those where the load identification algorithm failed to relate the extracted features to any of the library load features, and hence returned a null matrix.

Scenario 1 accurately detects and identifies all the cases and yields 100% for all the four considered performance evaluation metrics, and hence does not require further evaluation. However, as observed from the confusion matrices, the class is now unbalanced, as the number of null cases is equal to the sum of the number of all the test cases for eight loads. Therefore, for an unbiased evaluation, the macro-averaged F-measure $f_{\mathrm{mac}}$ and the weighted-averaged F-measure $f_{w}$ are calculated for scenarios 2 and 3.

**Figure 13:**
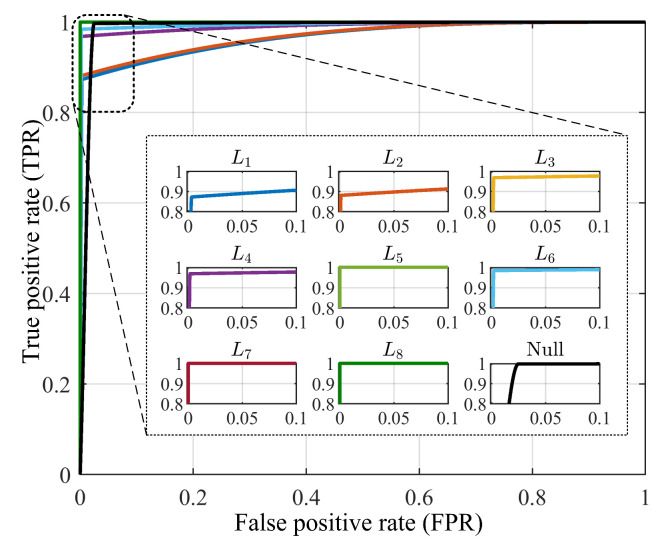
ROC curve for the proposed CRuST NILM.

To provide a graphical representation of the performance of the proposed NILM technique, the receiver operating characteristics (ROC) curve is plotted as in Figure 13. For this purpose, the true positive rates (TPR) and the false positive rates (FPR) are computed for each load case including the null case from the confusion matrix of the three scenarios. From the plots, it is observed that TP and TN do not overlap at all for loads $L_{5}$, $L_{7}$, and $L_{8}$, indicating 100% accuracy of event detection and load identification. The remaining set of loads and the null condition also yield very good performance as the area under the ROC curve (AUC-ROC) is close to unity as can be seen in Table 5 and Table 6. This validates the superior performance of the proposed CRuST NILM technique.

### 3.4. Comparison of Proposed CRuST NILM with Feed-Forward Back-Propagation Network Model

**Figure 14:**
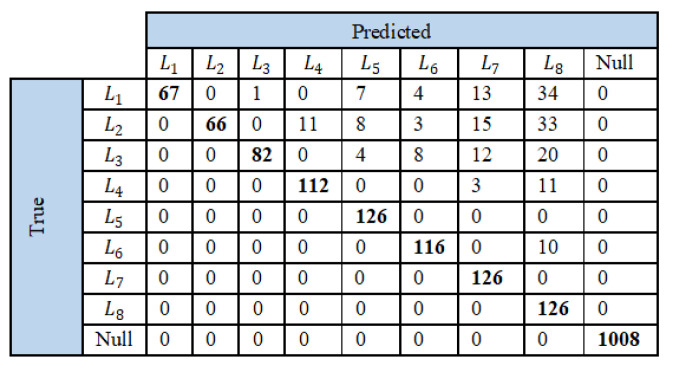
Confusion matrices for BPNM.

A feed-forward back-propagation network model (BPNM) was created to further validate and compare with the proposed CRuST NILM. The features of maximum current, THD, and crust factor for the laboratory dataset are obtained for all possible combinations of the eight loads. The resulting data matrix is split into a 3:2 ratio for training and validation, respectively. The validation set is later modified such that it remains identical to that used in CRuST NILM. The results obtained for BPNM in validation are presented in Figure 14 and Table 7.

For ease of direct comparison, the performance evaluation metrics obtained for CRuST NILM (given in Table 5 and Table 6) and BPNM (given in Table 7) are presented in terms of percentage values in Table 8. Among the PEMs, precision defines how accurate the predicted positive values are. It is noticed from Table 8, that the precision values obtained when CRuST NILM is used for loads $L_{1}$ through $L_{4}$ and null are lower than that obtained for BPNM. This is predominantly due to the narrow bands of separation criteria used in CRuST NILM for loads with low power consumptions. It can be improved by defining a wider separation criteria for the particular features that are a function of load power, thereby reducing false positives. For the remaining set of loads, CRuST NILM is observed to provide higher precision in comparison to BPNM. Hence, the macro-averaged and weighted-averaged precision for CRuST NILM is higher in comparison. Furthermore, CRuST NILM has superior performance compared with that of BPNM for all the loads $L_{1}$ through $L_{8}$ when considering the PEMs such as recall, F-measure, and accuracy.

### 3.5. Comparison of Proposed CRuST NILM with Other State of Art NILM Techniques

Finally the performance of the CRuST NILM technique developed in this paper is compared with other state of the art NILM techniques given in [21,24,25]. Direct comparison with other existing NILM techniques is difficult since the dataset used and the set of appliances used are different in each of the literature. Thus, we consider the $f_{1}$ score as a measure of overall NILM technique accuracy in order to make some comparison of the present NILM technique with the existing techniques mentioned above. Table 9 compares the average $f_{1}$ score obtained during the load identification phase for each NILM technique; alongside, the other input parameters such as the number of loads and input signal used in each NILM technique are also listed in the table.

Table 9 shows that the $f_{1}$ score obtained for the proposed load identification is 98%, which is an enhanced value compared with that of the other state of the art NILM techniques where the $f_{1}$ values read approximately as 77%, 97%, and 90%, respectively. This is predominantly due to the narrow bands of separation criteria used in CRuST NILM for the accurate detection and identification of the low-power appliances.

## 4. Conclusions and Future Work

This paper presented a NILM technique that uses current waveform features for a rule-based event detection and load identification of type 1 loads. Since current waveforms are load-specific, six features that signify the magnitude, phase, and waveshape are used to distinguish between the different loads. The appliance model consists of, for each feature, a load set array within which loads are grouped into sets based on the rules in criteria arrays. Furthermore, an event detection algorithm is presented that identifies a change in load using an event variable that is a function of the RMS and THD values of the change in current. In the load identification stage, the concept of set theory is employed to reduce the complexity and improve the accuracy of the algorithm. The results for event detection, load identification, and the combined proposed technique show superior performance with an accuracy greater than 96% for all the loads. The overall performance of CRuST NILM is observed to be superior when compared with that of a feed-forward BPNM for NILM and with other state of the art NILM techniques.

As part of future work, the proposed algorithm will be improved to include type 2 and 3 loads by using state transition tables/diagrams. Additionally, the appliance model will adapt a dynamic library such that when the load identification stage returns a null set, the features are saved on to a temporary library to identify their recurrence and thereby modify the dynamic library. In this manner, unsupervised learning will be enabled in the future work related to the proposed NILM.

## Figures and Tables

**Table 1:** Loads, labels, and features of their current waveforms.

Load	Label	Crest Factor	Form Factor	Variance (%)	RMS (A)	PF	THD (%)
	$L_{n}$	*C*($L_{n}$)	*F*($L_{n}$)	*V*($L_{n}$)	*R*($L_{n}$)	*P*($L_{n}$)	*T*($L_{n}$)
CFL1	$L_{1}$	3.06	0.975	49.89	0.050	0.945	99.1
		3.07	0.972	50.12	0.049	0.946	99.6
CFL2	$L_{2}$	2.95	1.008	45.67	0.110	0.964	89.9
		2.96	1.008	45.97	0.108	0.963	90.5
Bulb1	$L_{3}$	1.39	1.108	0.06	0.301	0.674	2.03
		1.39	1.108	0.06	0.301	0.675	2.04
Bulb2	$L_{4}$	1.38	1.108	0.09	0.456	0.613	2.14
		1.38	1.109	0.10	0.456	0.613	2.13
Fan	$L_{5}$	1.70	1.148	6.96	0.596	0.719	24.4
		1.70	1.148	6.90	0.600	0.720	24.2
Desktop	$L_{6}$	3.08	1.004	48.61	0.237	0.820	95.8
		3.09	1.004	48.67	0.237	0.820	95.9
Fridge	$L_{7}$	1.39	1.105	0.07	6.021	0.565	1.93
		1.39	1.106	0.06	6.026	0.565	1.92
Heater	$L_{8}$	1.40	1.110	0.03	9.313	0.568	1.52
		1.40	1.109	0.03	9.309	0.569	1.51

**Table 2:** Performance evaluation of event detection algorithm.

Parameter	Scenario 1	Scenario 2	Scenario 3
Precision	1	0.99549	0.99698
Recall	1	0.98661	0.98313
F-measure	1	0.99142	0.98999
Accuracy	1	0.98222	0.98022

**Table 3:** Performance evaluation of load identification for scenario 2.

PEM	$L_{1}$	$L_{2}$	$L_{3}$	$L_{4}$	$L_{5}$	$L_{6}$	$L_{7}$	$L_{8}$
TP	21	21	26	25	28	27	28	28
FP	6	4	3	1	1	0	0	0
FN	4	7	2	3	0	1	0	0
TN	190	189	190	192	192	193	193	193
*P*	0.8750	0.8846	0.8966	0.9615	0.9655	1.0000	1.0000	1.0000
*R*	0.8400	0.8214	0.9286	0.8929	1.0000	0.9643	1.0000	1.0000
$f_{1}$	0.8571	0.8519	0.9123	0.9259	0.9825	0.9818	1.0000	1.0000
*Acc*	0.9679	0.9636	0.9774	0.9819	0.9955	0.9955	1.0000	1.0000

**Table 4:** Performance evaluation of load identification for scenario 3.

PEM	$L_{1}$	$L_{2}$	$L_{3}$	$L_{4}$	$L_{5}$	$L_{6}$	$L_{7}$	$L_{8}$
TP	110	111	122	122	126	124	126	126
FP	6	2	4	2	0	2	0	0
FN	7	9	3	4	0	1	0	0
TN	868	869	862	863	865	864	865	865
*P*	0.9483	0.9823	0.9683	0.9839	1.0000	0.9841	1.0000	1.0000
*R*	0.9402	0.9250	0.9760	0.9683	1.0000	0.9920	1.0000	1.0000
$f_{1}$	0.9442	0.9528	0.9721	0.9760	1.0000	0.9880	1.0000	1.0000
*Acc*	0.9869	0.9889	0.9929	0.9939	1.0000	0.9970	1.0000	1.0000

**Table 5:** Performance evaluation of CRuST NILM for scenario 2.

PEM	$L_{1}$	$L_{2}$	$L_{3}$	$L_{4}$	$L_{5}$	$L_{6}$	$L_{7}$	$L_{8}$	Null
TP	21	23	26	25	28	27	28	28	223
FP	3	3	3	1	1	1	0	0	7
FN	7	5	2	3	0	1	0	0	1
TN	417	417	417	419	419	419	420	420	217
*P*	0.88	0.88	0.9	0.96	0.97	0.96	1.00	1.00	0.97
*R*	0.75	0.82	0.93	0.89	1.00	0.96	1.00	1.00	1.00
$f_{1}$	0.81	0.85	0.91	0.93	0.98	0.96	1.00	1.00	0.98
*Acc*	0.98	0.98	0.99	0.99	1.00	1.00	1.00	1.00	0.98
$f_{\mathrm{mac}}$	0.94								
$f_{w}$	0.96								

**Table 6:** Performance evaluation of CRuST NILM for scenario 3.

PEM	$L_{1}$	$L_{2}$	$L_{3}$	$L_{4}$	$L_{5}$	$L_{6}$	$L_{7}$	$L_{8}$	Null
TP	110	111	122	122	126	124	126	126	1005
FP	6	2	4	3	0	4	0	0	25
FN	16	15	4	4	0	2	0	0	3
TN	1884	1888	1886	1887	1890	1886	1890	1890	983
*P*	0.95	0.98	0.97	0.98	1.00	0.97	1.00	1.00	0.98
*R*	0.87	0.88	0.97	0.97	1.00	0.98	1.00	1.00	1.00
$f_{1}$	0.91	0.93	0.97	0.97	1.00	0.98	1.00	1.00	0.99
*Acc*	0.99	0.99	1.00	1.00	1.00	1.00	1.00	1.00	0.99
$f_{\mathrm{mac}}$	0.97								
$f_{w}$	0.98								
AUC-ROC	0.98	0.98	0.99	0.99	1.00	0.99	1.00	1.00	0.99

**Table 7:** Performance evaluation of BPNM.

PEM	$L_{1}$	$L_{2}$	$L_{3}$	$L_{4}$	$L_{5}$	$L_{6}$	$L_{7}$	$L_{8}$	Null
TP	67	66	82	112	126	116	126	126	1008
FP	0	0	1	1	19	15	43	109	0
FN	59	60	44	14	0	10	0	0	1
TN	1891	1890	1889	1889	1871	1875	1847	1782	1890
*P*	1.00	1.00	0.99	0.99	0.87	0.89	0.75	0.54	1.00
*R*	0.53	0.52	0.65	0.89	1.00	0.92	1.00	1.00	1.00
$f_{1}$	0.69	0.69	0.78	0.94	0.93	0.90	0.85	0.70	1.00
*Acc*	0.97	0.97	0.98	0.99	0.99	0.99	0.98	0.95	1.00
$f_{\mathrm{mac}}$	0.83								
$f_{w}$	0.90								

**Table 8:** Comparison of PEMs for proposed CRuST NILM and BPNM.

PEM	Method	$L_{1}$	$L_{2}$	$L_{3}$	$L_{4}$	$L_{5}$	$L_{6}$	$L_{7}$	$L_{8}$	Null	Macro-Avg	Weighted-Avg
*P*	CRuST	95.00	98.00	97.00	98.00	100.00	97.00	100.00	100.00	98.00	98.11	98.06
	BPNM	100.00	100.00	99.00	99.00	87.00	89.00	75.00	54.00	100.00	89.22	93.94
*R*	CRuST	87.00	88.00	97.00	97.00	100.00	98.00	100.00	100.00	100.00	96.33	97.94
	BPNM	53.00	52.00	65.00	89.00	100.00	92.00	100.00	100.00	100.00	83.44	90.69
$f_{1}$	CRuST	91.00	93.00	97.00	97.00	100.00	98.00	100.00	100.00	99.00	97.22	98.00
	BPNM	69.00	69.00	78.00	94.00	93.00	90.00	85.00	70.00	100.00	83.11	90.50
Acc	CRuST	99.00	99.00	100.00	100.00	100.00	100.00	100.00	100.00	99.00		
	BPNM	97.00	97.00	98.00	99.00	99.00	99.00	98.00	95.00	100.00		

**Table 9:** Comparison of CRuST NILM with existing NILM techniques.

NILM Technique	Load Count	Input Signal	F-Measure (%)
State of art model [25]	3	Current harmonics	97
State of art model [21]	11	Current and voltage	90
State of art model [24]	7	Power	77
Proposed technique	8	Current	98

## Data Availability

Not applicable.

## Abbreviations

The following abbreviations are used in this manuscript:

AUC	Area under curve
BPNM	Back-propagation network model
CFL	Compact fluorescent lamp
CRuST	Current waveform features with rule-based set theory
FPR	False positive rates
NILM	Non-intrusive load monitoring
PF	Power factor
PEM	Performance evaluation metrics
PSO	Particle swarm optimization
RMS	Root-mean-square
ROC	Receiver operating characteristics
THD	Total harmonic distortion
TP	True positive
TN	True negative
TPR	True positive rates
